# TSCH and RPL Joining Time Model for Industrial Wireless Sensor Networks

**DOI:** 10.3390/s21113904

**Published:** 2021-06-05

**Authors:** Jose Vera-Pérez, Javier Silvestre-Blanes, Víctor Sempere-Payá

**Affiliations:** 1 Instituto Tecnológico de Informática, 46022 Valencia, Spain; 2 ITI and Departamento de Informática de Sistemas y Computadores (DISCA), Universitat Politècnica de València (UPV), 03801 Alcoy, Spain; jsilves@disca.upv.es; 3 ITI and Departamento de Comunicaciones (DCOM), Universitat Politècnica de València (UPV), 46022 Valencia, Spain; vsempere@dcom.upv.es

**Keywords:** WSN, industrial internet of things, synchronization, TSCH, RPL

## Abstract

Wireless sensor networks (WSNs) play a key role in the ecosystem of the Industrial Internet of Things (IIoT) and the definition of today’s Industry 4.0. These WSNs have the ability to sensor large amounts of data, thanks to their easy scalability. WSNs allow the deployment of a large number of self-configuring nodes and the ability to automatically reorganize in case of any change in the topology. This huge sensorization capacity, together with its interoperability with IP-based networks, allows the systems of Industry 4.0 to be equipped with a powerful tool with which to digitalize a huge amount of variables in the different industrial processes. The IEEE 802.15.4e standard, together with the access mechanism to the Time Slotted Channel Hopping medium (TSCH) and the dynamic Routing Protocol for Low-Power and Lossy Networks (RPL), allow deployment of networks with the high levels of robustness and reliability necessary in industrial scenarios. However, these configurations have some disadvantages in the deployment and synchronization phases of the networks, since the time it takes to synchronize the nodes is penalized compared to other solutions in which access to the medium is done randomly and without channel hopping. This article proposes an analytical model to characterize the behavior of this type of network, based on TSCH and RPL during the phases of deployment along with synchronization and connection to the RPL network. Through this model, validated by simulation and real tests, it is possible to parameterize different configurations of a WSN network based on TSCH and RPL.

## 1. Introduction

The concept of Industry 4.0 has revolutionized industry as we know it today, adding new degrees of digitalization to plant processes and providing greater knowledge of the different phases of the value chain. The new information obtained through digitalization, together with different technologies such as artificial intelligence, machine learning or analytical techniques based on big data, allow more precise optimization of the different processes involved in the value chain. It also allows the operator to perform low-level monitoring and even to carry out estimates, which allow predictive maintenance techniques to be carried out, to detect possible errors before they even occur [1].

However, this Industry 4.0 infrastructure requires a massive digitalization upgrade process from the current industry, so that the digitalization of new variables provides information that until now went unnoticed. Among all the technologies capable of carrying out this process, wireless sensor networks (WSNs) are positioned as one of the most relevant, thanks to their low consumption characteristics and their easy scalability when providing communication to a large number of sensors. This allows the deployment of large networks at a low cost.

There are many technologies through which to carry out the deployment of a WSN, for example, Bluetooth and IEEE 802.15.4 for the personal area networks or LoRaWAN and SigFox for wide area networks, among others. However, industrial scenarios are characterized by a more aggressive means of communication in terms of multi-path fading, which means that not all technologies are capable of meeting the minimum operating requirements. The IEEE 802.15.4 [2] standard, together with the Time Slotted Channel Hopping (TSCH) mechanism, is specifically designed to work in industrial scenarios [3], whose requirements are characterized by high reliability, low latency and a maximum number of devices between 10 and 30 nodes, depending on the field of application. For these reasons, the TSCH mechanism is one of the most widely used solutions as a medium access control (MAC) mechanism in industrial environments. The main characteristics by which TSCH stands out against other access mechanisms defined in the IEEE 802.15.4 standard are the deterministic behavior of its scheduling in time slots and the technique of frequency hopping, which allows it to mitigate the possible interference or fading that occurr in a radio channel.

Regarding the formation of ad hoc mesh networks and the creation of routes between the different nodes that form the network, the IPv6 Routing Protocol for Low-Power and Lossy Networks (RPL) [4] has become one of the most common solution [5], Ref. [6] for the creation of dynamic routes in WSN networks with few resources. Together with the IEEE 802.15.4e standard, they allow the deployment of robust WSNs, creating multi-hop mesh networks with support for IPv6-based networks. Although the use of both protocols together allows deployment of robust networks with a deterministic behavior during the stationary state, the deployment and synchronization phase may present some difficulties. This is mainly due to the operation of the TSCH mechanism. The nodes already synchronized will be sending the necessary messages to establish synchronization with the new nodes using different frequency channels depending on the instant in which the message is being sent. This behavior makes it more complex for the new nodes to receive the information needed to synchronize with the TSCH network, and later join the RPL network, since they do not know the instant of time or on which channel the synchronization message will be transmitted.

This particular drawback of TSCH-based networks has been researched over the past few years, and different approaches have been proposed to reduce synchronization and connection times, since the standard does not propose any optimized configuration for certain application domains. However, the relationship between the TSCH medium access mechanism and the RPL protocol has not been studied as deeply as TSCH synchronization, but their relationship is particularly important, since they are part of the complete process of deploying a new node. An analytical model both for TSCH and RPL has been proposed in this article, allowing the characterization of the total time that a node takes to connect to a network based on TSCH andn RPL. Using this model it is possible to choose an optimal configuration, both for TSCH and RPL, that allows one to improve TSCH synchronization and DODAG RPL connection times. Depending on the use case of the WSN, this model allows to obtain an optimized configuration in each case, focusing on improving the network formation time or extending the whole battery lifetime of the network.

The rest of the article is structured as follows. Section 2 gives a brief introduction to the TSCH access mechanism, which is part of the IEEE 802.15.4 standard, and also of the RPL dynamic routing protocol, emphasizing those aspects that are most relevant during the deployment, RPL tree formation and joining of new nodes to the network phases. In Section 3, an analysis of the current state of the art is presented, reviewing those works that have evaluated different proposals for improvements in the processes of synchronization and joining in networks based on TSCH and RPL. In Section 4 and Section 5, the proposed analytical modeling is detailed, for both TSCH and RPL, respectively. Section 6 describes the different simulation scenarios that have been proposed to perform the validation of the analytical model, showing the results obtained for each of them. A series of tests with real equipment were also carried out to validate the results obtained in the simulations, the results of which are shown in Section 7. Finally, Section 8 presents the conclusions drawn from this analysis.

## 2. TSCH and RPL Network Deployment

The process of forming a network based on TSCH and RPL begins with a first phase in which the nodes that have just been turned on need to receive an Enhanced Beacon (EB) message from nodes already synchronized. This message contains the necessary information for the new nodes to synchronize with the rest of the network. After this first phase of synchronization, the scheduling defined in TSCH is used to carry out the exchange of RPL signaling messages. This RPL message exchange allows the new node to join the topology, allowing other nodes further away to determine the route to follow to reach the newly joined node, both for downstream and upstream traffic. There follows a detailed description of the operation of these two mechanisms, which will help to define the analytical model that characterizes them.

### 2.1. TSCH Beacon Advertising

In the first revisions of the standard, different access mechanisms were proposed to improve the performance of networks based on IEEE 802.15.4, such as DSME (Deterministic and Synchronous Multi-channel Extension), LLDN (Low Latency Deterministic Network) and TSCH (Time Slotted Channel Hopping). Among these mechanisms, TSCH stands out, as it focuses on the deployment of networks in industrial scenarios, allowing a high degree of reliability and robustness to be achieved thanks to its time synchronization and techniques to mitigate interference and fading.

The access mechanism to the TSCH medium allows the temporal plane to be structured in such a way that a certain number of slots are repeated periodically over time. This subset of slots is called a slotframe, and its length determines how often the same slot is repeated. In addition to time synchronization, the TSCH mechanism uses a frequency hopping technique to mitigate interference, which allows the use of a different transmission channel for each time slot in which communication is established between two nodes. The use of a different channel to send the information between two nodes allows us to compensate for any interference or fading that may be occurring in a certain channel, which improves the robustness and reliability of communications. The following equation is used to calculate the channel used in each timeslot:

(1) $$f=F\{\left(ASN+ch_{offset}\right)\cdot mod\left(C\right)\}$$

where *f* is the physical transmission channel; ASN is the absolute slot number, which corresponds to the number of slots that have passed since the network was deployed; the $ch_{offset}$ parameter allows for different channels to be used in the same time slot; and, *C* corresponds to the total number of channels that are used. Therefore, the function F{−} consists of selecting a channel of a predefined sequence according to the index that is obtained after calculating the module.

The scheduling of these radio resources (timeslot and frequency channels) allows the exchange of information between the network nodes to be optimized. This is because it is possible to configure a contention free scheduling, with high immunity to interferences, thanks to frequency hopping, and with high energy savings due to the fact that slots that are not used are totally disabled. The method to define this scheduling is not described by the standard, but some recommendations have been proposed to allocate minimum resources to carry out this exchange of messages between nodes, known as minimal 6TiSCH configuration [7,8]. Different scheduling mechanisms focused on TSCH have also been previously proposed, such as the autonomous planning mechanism Orchestra [9]. This mechanism allows the nodes to build the TSCH schedule autonomously, without the need for additional signaling traffic, and allows the schedule to be separated into different traffic planes.

Since the time plane is structured in timeslots, it is necessary that the nodes belonging to the same TSCH network are synchronized so that the exchange of messages occurs simultaneously between two nodes. To achieve this, it is necessary to perform a previous phase of synchronization, in which the new nodes that join the network must obtain the information on the scheduling used, as well as adjust the time base to a reference of the nodes that already belong to the network.

The nodes that already belong to the network and are correctly synchronized send periodic signaling messages with information about the schedule used and the current value of the ASN parameter. This is necessary to carry out the synchronization process. These signaling messages are known as Enhanced Beacons, an improved version of the traditional beacons proposed by the IEEE 802.15.4 standard, which allow additional information to be added in the fields known as Information Elements.

**Figure 1:**
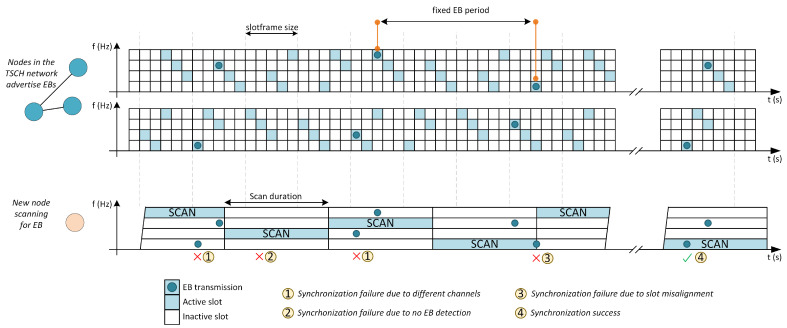
Example of beacon advertising process using four channels.

On the other hand, the nodes that want to join the network start a scanning process. In this process, the nodes go through the different pre-configured channels, for a certain period of time, until they receive an EB correctly. This synchronization process will take longer depending on different factors such as the total number of configured channels, the time each channel is scanned or the transmission period of the EB message. Figure 1 shows a representation of the main causes that can lengthen this synchronization process, such as no Enhanced Beacon being sent during scanning time or the channel being scanned not matching the channel on which the Enhanced Beacon message is being transmitted.

### 2.2. RPL Tree Network Topology Construction

Once the nodes have managed to synchronize with the TSCH network, they have the necessary information to be able to establish communication with the rest of the nodes in the network. However, they do not know which nodes already belong to the network, nor how to reach those nodes that are not within their coverage area. For this reason, it is necessary to build a network topology in order to be able to establish communication in the multi-hop network. Wireless sensor networks are made up of devices with reduced computing capabilities and low power consumption. The RPL protocol calls these types of networks low-power lossy networks. This protocol is designed to be a lightweight routing protocol with low computing requirements, making it ideal for deployment in WSNs. For this reason, the RPL protocol is widely used in network deployments based on the IEEE 802.15.4 standard, including those using TSCH, thus allowing routes to be established between devices and maintaining a meshed network topology.

To maintain the network topology in situations where wireless links can vary continuously, the RPL protocol uses a series of objective functions, which allow the nodes to update their routes dynamically and autonomously. These objective functions are based on monitoring the connection quality through different metrics, allowing the nodes to change their routes if the current connection quality is degraded [10]. For the formation of the RPL tree, known as DODAG (Destination Oriented Directed Acyclic Graph), different control messages of the type ICMPv6 are used. The DODAG Information Object (DIO) messages are sent by the nodes that are already part of the network, in order to keep the routes updated and allow new nodes to obtain the RPL topology information. DIO messages can be sent either by multi-cast, triggered by the Trickle Timer, or unicast methods in response to an explicit request. DODAG Information Solicitation (DIS) messages are sent to request nodes that already belong to the network to send a DIO message with the RPL tree information. Finally, DAO (Destination Advertisement Object) messages allow paths to the RPL root node to be created. This DAO message is sent through all nodes in the path between the node that originates the DAO message and the root.

**Figure 2:**
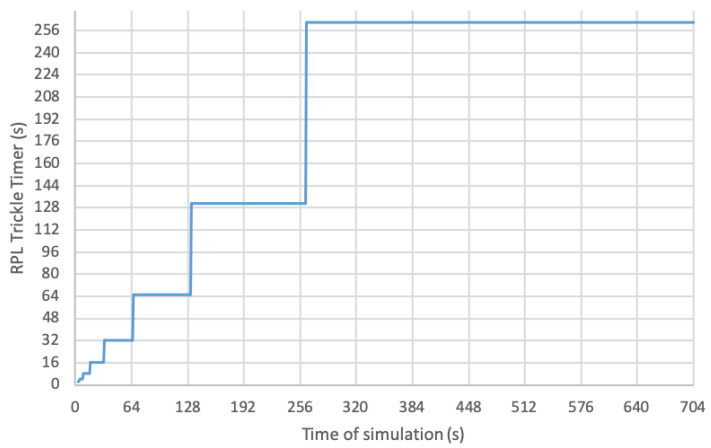
RPL Trickle Timer evolution in time for $I_{min}=4$ s and *Doublings times* = 6.

The RPL protocol uses a timer to mark the sending of signaling traffic. This is a timer that increases its period over time, and its operation is based on a Trickle Timer, whose operation is described in RFC6206 [11]. The use of this type of timer to send DIO messages allows this traffic to be reduced once the network is deployed and stable, reducing the congestion that signaling traffic can produce. The algorithm allows the value of the DIO Trickle Timer to be increased as long as the information exchanged by the different nodes in the topology does not indicate that any change has occurred that requires an update. This timer will restart whenever any inconsistency is detected in the RPL topology, such as in the case of a parent change, the loss of a network node or also due to the reception of a DIS message. Figure 2 shows the evolution of the RPL Trickle Timer. Every time the configured timer expires (starting with an initial value of $I_{min}=4$ s), the algorithm doubles its value and plans a new transmission of a DIO message, repeating this process until the timer reaches the maximum configured value.

In the specific case shown in the graph, the Trickle Timer reaches its maximum value in a few minutes, causing a DIO message to be sent once every few minutes thereafter. The authors of [12] carry out a detailed analysis showing the evolution of the RPL Trickle Timer. The Trickle Timer state can only be reset if a DIS message is received, starting the algorithm again with its minimum value, as seen in Figure 2. In implementations such as Contiki, it is proposed to use the current state of the Trickle Timer to modify the value of the EB transmission period, since it allows speeding up the synchronization process when the value of the Trickle Timer is low, in addition to avoiding congesting the network with EB messages when the Trickle Timer value is high. This behavior directly affects the process of deploying a new node, since if the nodes that make up the network have a very high Trickle Timer value, the EB period will also be high and the new nodes will take longer to obtain the synchronization to the TSCH network. In this situation, it is not possible to reset the Trickle Timer value using DIS messages, since the new node waiting to join the network is not yet synchronized.

## 3. Related Work

Over the past few years, since the TSCH media access mechanism was introduced in the 2012 IEEE 802.15.4 standard, research papers have been submitted, researching different ways to improve the synchronization processes for TSCH. The authors of [13,14,15,16] propose different frameworks and configurations for scheduling in TSCH in order to mitigate the synchronization problem, so as to improve connection times based on enabling more radio resources for sending signaling messages. Vogli et al. [13] propose two different frameworks for TSCH scheduling, improving the network connection processes through the use of random slots for sending periodic EBs. Duy et al. [14] propose a new framework by which to determine the optimal number of EBs needed to establish synchronization quickly. Khoufi et al. [15] make a comparison with the state of the art of a new framework that allows the TSCH beacons to be sent through all channels of the network, ensuring that no collisions will occur, and thus improving on the results obtained in [13]. The authors of [16] define a different framework that allows the nodes to estimate the best channel through which to transmit the signaling messages, so their solution is focused on particularly noisy scenarios.

Karalis et al. [17,18] carried out two studies that led to an improvement in the synchronization times in TSCH networks, firstly, by proposing a schedule for the nodes to send beacons in slots that are free of collisions, also considering that these nodes can be mobile nodes, and secondly, increasing the traffic of EB messages without adding new slots, sending several EB messages in different channels using a single timeslot. De Guglielmo et al. [19] conducted a study on how to improve the synchronization process in situations where a beacon sending mechanism with a random period is used, showing how synchronization times improve with the influence of other parameters such as the number of channels used by the WSN network. Wang et al. [20] conducted a study similar to those previously mentioned, in which they performed different simulations modifying the density of nodes in the network and the period of transmission of EB messages, showing that, as in the article [19], a random transmission period improves the synchronization process in TSCH. In [21], the authors propose a mechanism that allows modification of the beacon transmission period according to the number of nodes in the network, thus reducing congestion in networks with higher node density.

The above studies focus on analyzing only the TSCH synchronization process. However, in [22] the authors consider the complete communications stack, and compare its dynamic resource management algorithm for 6TiSCH networks with the state of the art, demonstrating that it improves the synchronization processes in TSCH networks. In [23] a complete protocol stack is proposed, using both TSCH and RPL in order to achieve high reliability and robustness for industrial scenarios. To reduce the network formation times, the authors propose a simple mechanism that reduces the slotframe size during the network formation period. However, no information is given on how long the network formation takes or the energy impact cased by the increase in EB messages. The authors of [24,25] carried out a study in which they validate how the synchronization time improves in TSCH-RPL networks by using a dynamic EB transmission period, proposing a cyclic algorithm that they call Bell-X, with windows of opportunity in which the probability of a node connecting rapidly increases.

However, although these studies propose mechanisms that improve the synchronization processes, they are rarely supported by an analytical model that allows characterization of the synchronization and RPL joining processes, which can help to determine the most optimal configuration for a parameterizable algorithm. Yaala et al. [26] have developed an analytical model to characterize the synchronization processes of TSCH networks using slots shared by the network nodes and with sporadic traffic, validating the model through simulations. The authors of [22] also support their study by developing an analytical model for both the TSCH synchronization and the RPL Trickle Timer.

This paper takes the results obtained in [25] as a starting point, proposing an analytical model to characterize the processes of synchronization in TSCH and connection with the RPL topology, so that, with this tool, it is possible to choose more optimal configurations when choosing a period of transmission of EB messages with dynamic behavior.

## 4. TSCH Analytical Model

The objective of the analytical model is to be able to correctly estimate the average time that the synchronization process can take for a new node to join the network, and in the same way to estimate the maximum time that the formation of a complete network will take. Furthermore, knowing the schedule, it is possible to estimate the consumption of the nodes, since each platform has an estimated consumption for the transmission/reception processes. The synchronization process will be affected by the following parameters:

- EB transmission period ($T_{EB}$). This period marks the time when a TSCH beacon is generated. Its value does not have to coincide with the size of the slotframe, since this message is sent in the next active slot once the beacon has been generated. The beacon transmission period can improve the processes of synchronization and deployment of the network by following a dynamic adjustment of the period, as demonstrated by the bell-shaped pattern in article [25].
- Scanning period ($T_{SCAN}$). This period marks the time that each channel is scanned individually. Nodes that want to join the network listen to the same channel, configuring channels at random and repeating this process until an EB message is received by the node.
- Number of neighbors (*N*). The number of nodes that are within the coverage area of the node that wants to join. Being within the coverage area, the node that joins the network will be able to receive EB messages from the N nodes.
- Number of channels (*C*). This represents the subset of channels used, both in the normal operation of the network and in the scanning phase. There are a total of 16 channels in the 2.4 GHz band, but a subset can be selected to limit the channels used during normal network operation. This number of channels will affect the synchronization time as the new node must search a larger number of channels to find a beacon to synchronize with.
- Packet Delivery Rate (PDR). This parameter represents the probability of success of receiving a packet correctly. Signaling messages may be lost due to a collision with another message or due to interference in the transmission channel.

Table 1 summarizes these main variables along with other parameters that influence the synchronization process.

Starting from a schedule where a slotframe of size $SF_{SIZE}$ is configured, each node that is part of the network is assigned a guaranteed slot for beacon transmission thanks to the help of the autonomous scheduler Orchestra. This slot will be assigned from the node identifier, so that a maximum of $SF_{SIZE}$ nodes can join the network using a guaranteed slot for the same channel offset. In this way, it is ensured that there are no collisions between beacon messages.

Assuming that the scanning period $T_{SCAN}$ is high enough ($T_{SCAN}>>T_{EB}$) not to change channels until a beacon is correctly received, we can calculate the average number of slotframes needed to correctly receive a beacon. If a node is listening on a fixed channel 100% of the time, at most it will be necessary to send *C* beacons if a different channel is used in each slotframe. To get the same slotframe to use different channels, it is necessary to configure a $SF_{SIZE}$ with a prime value [27].

This average number of slotframes needed to receive a beacon correctly (SF) can be modeled as a uniform discrete random variable between 1 and *C*. This will mean that, at best, the first beacon sent matches the scan channel, requiring only one slotframe. In the worst case, *C* slotframes will be needed to receive a beacon on any of the configured channels. All intermediate cases can occur with the same probability. If it is also taken into account that it is possible to have losses due to the channel, the average number of slotframes would be represented by the following equation:

(2) $$\bar{SF}=\frac{C+1}{2}\cdot \frac{1}{PDR}$$

Assuming that a beacon is sent with a transmission period of $T_{EB}$, this factor would multiply the average number of slotframes. In the case that there are *N* nodes sending beacons within the coverage area of the node trying to join, the average value of beacon transmission period can be understood as $T_{EB}/N$, since *N* beacons are sent within the same slotframe. The average time needed for a node to synchronize, when sending a message with a fixed transmission period of $T_{EB}$ would be represented by the following equation:

(3) $$T_{SYNC}=\frac{T_{EB}}{N}\cdot \frac{C+1}{2}\cdot \frac{1}{PDR}$$

## 5. RPL Analytical Model

In the case of RPL, the nodes must already be synchronized by TSCH, and they need to obtain the information from the RPL tree. To do this, they must receive a DIO message from one of the nodes in the network, within their coverage area, and then send a DAO to the root. This DAO message will influence the connection time with the RPL tree, since the messages must be transmitted several hops away from the root.

RPL and TSCH schedules must not overlap, so we will be able to avoid the collisions that could occur between these two sources of traffic. This consideration is achieved thanks to the use of Orchestra when obtaining the TSCH schedule. This allows the creation of two schedules for each traffic plane, while avoiding collisions between them. In addition, for the RPL traffic plane, the system starts from a scheduling based on minimal 6TiSCH, in which only one slot shared by all the nodes that are part of the network is enabled, so that access to the radio resource will be resolved through contention. Table 2 shows the different parameters that influence the process of joining the RPL topology. These parameters have been taken into account when defining the analytical model, which characterizes the time it takes a node to obtain this configuration.

After the synchronization phase, the nodes that are already part of the RPL tree send DIO messages based on the current value of their Trickle Timer. From this, the probability of a node transmitting a DIO message is established.

(4) $$P_{dio}=\frac{SF_{RPL}}{T_{trickle}}$$

This probability will be determined by the size of the slotframe, $SF_{RPL}$, which will only have one shared active slot, and also by the current value of the Trickle Timer, $T_{trickle}$, which will be different depending on when the Trickle Timer is found, or if it is restarted, since the model seeks to characterize each of the individual states of the Trickle Timer, represented by the steps in Figure 2. To avoid collisions within this shared slot, it is possible to establish the probability of this happening, which will be:

(5) $$P_{dio}\cdot {\left(1-P_{dio}\right)}^{N-1}$$

where N−1 is the number of nodes that are not sending DIO messages. As we can receive messages from all the nodes, this probability will be multiplied by *N*.

(6) $$N\cdot P_{dio}\cdot {\left(1-P_{dio}\right)}^{N-1}$$

By analyzing the probability of transmission success (PDR), it is possible to determine the number of messages, or number of slotframes to be received correctly. With a probability determined by the PDR, the DIO message will be received in the worst case at a time determined by the $SF_{RPL}$ variable, and in the best case, it will be received in the next timeslot. If its average value is taken, there will be a time equal to $SF_{RPL}/2$. With a probability (1−PDR), the DIO message will not be transmitted in the first slotframe, but in the following slotframes, there will be a maximum of four retries for each message.

(7) $$t_{pdr}=\sum_{i=0}^{4}\left(SF_{RPL}\cdot i+\frac{SF_{RPL}}{2}\right)\cdot PDR\cdot {\left(1-PDR\right)}^{i}$$

The transmission time of a DIO correctly sent will be as follows, where $T_{trickle}/2N$ is the average time to receive a DIO message with *N* neighboring nodes, to which is added the time required to send a DIO, which depends on the PDR and on the possible collision with other DIO messages:

(8) $$T_{DIO}=\frac{T_{trickle}}{2\cdot N}+\frac{t_{pdr}}{N\cdot {\left(1-P_{dio}\right)}^{N-1}}$$

During the simulations carried out to validate the proposed RPL model, only the reception time of a DIO message was used, since it reflects the moment when the node itself obtains the routing information and becomes fully connected. To complete the process of creating routing tables for downstream traffic, it is necessary to send back a DAO message to the root node once the new node obtains the RPL information from the DIO message. This process has not been taken into account during the analysis of the simulations, since it is a process that takes places after the connection of the new node.

## 6. Simulations and Results

To carry out the validation of the proposed analytical model, both for the TSCH model and for the RPL model, different simulations were carried out. In these simulations the number of neighbors that already belong to the network and the transmission period of the signaling messages (beacons in the case of TSCH and the DIO messages in the case of the RPL protocol), were modified.

The configuration of the protocol stack, as well as the different variations of each of the simulated configurations, were based on the Contiki 3.0 operating system. In this operating system it is possible to configure a complete communications stack, based on IEEE 802.15.4, with different mechanisms for accessing the medium, including TSCH, and with protocols such as RPL for the routing and 6LoWPAN as an IPv6 adaptation layer. This is necessary to encapsulate IP information in IEEE 802.15.4 frames. It also has different applications that are useful in the operation of TSCH-based WSNs, such as the autonomous scheduling mechanism Orchestra, which is used in these simulations.

The development environment of Contiki has a simulator called Cooja, which allows simulation of the behavior of a real sensor network based on the applications developed in Contiki. Table 3 summarizes the main physical configuration parameters, which will be common to all the simulations performed in this study. As indicated in the table, each of the configurations will be repeated a total of 30 times to have a sufficient statistical mass with which to assess the validity of the analytical model.

**Figure 3:**
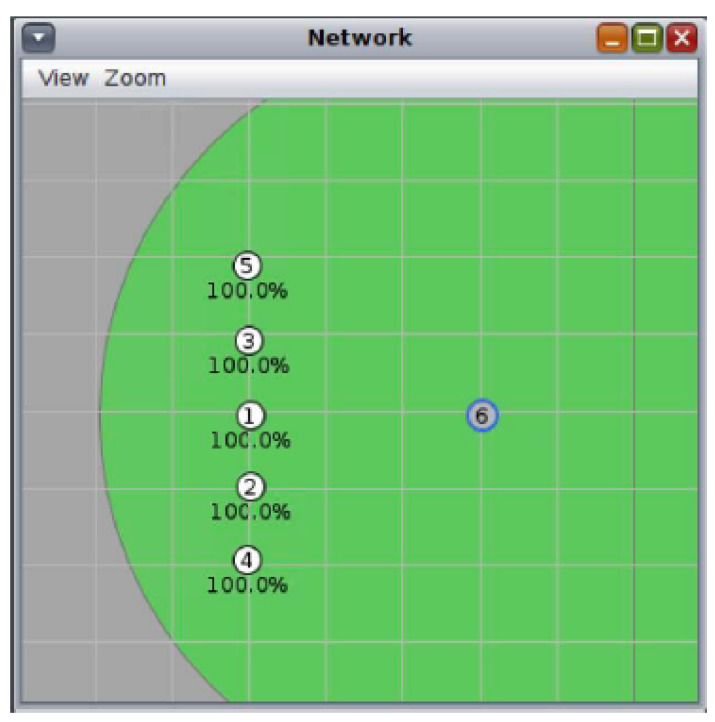
Network deployment in Cooja Simulator for N=5.

As can be seen in Figure 3, the nodes that will be part of the set of devices already synchronized are located in line, always within the coverage area of the new node that has been added—in this case, node 6. This deployment has been carried out because, although the physical network topology does not matter, it is necessary that all the nodes that are already connected to the network are within the coverage area of the new node in order to send signaling traffic from both TSCH and RPL.

The procedure to be followed in each simulation will start with the deployment of a network with a certain number of nodes, depending on the configuration to be characterized. Once all the nodes are connected, a time will be left for the network to stabilize and no changes in the topology will occur. This time will be between 15 and 20 min from when the network is started. Once this time has passed, a new node is added to the topology, which will be the node studied.

The two events that determine the evolution of the simulation are: the instant in which the new node synchronizes with the network when it receives a Beacon from TSCH and the instant in which the new node receives a DIO message to obtain the topology information. Table 4 shows the basic configuration of the protocol stack used in all the simulations. In the case of scheduling, an Orchestra-based scheduling was chosen, since it allows us to separate the scheduling into different traffic planes, giving priority to those messages that are more important. In addition, to prevent these schedules from overlapping, a slotframe size ($SF_{SIZE}$) has been configured equal to 101 slots in both cases. The size of 101 slots allows the same slot to repeat approximately every second, which allows for enough slots to transmit signaling messages every 4 s.

Two types of simulations were performed, one to characterize the evolution of the synchronization process in TSCH, and another to characterize the connection time of the new node to the RPL tree. In this way, it is possible to isolate the behavior of each of the protocols, so that changes in the configuration of the other protocol have as little influence as possible.

In the case of the simulations for TSCH, both the number of neighbors already synchronized and the period of beacon transmission were modified, allowing the behavior with different configurations to be observed. On the RPL side, its configuration was left as the default, leaving the Trickle Timer to modify the DIO transmission period. The simulation ends at the moment the new node receives the EB message in order to synchronize with the WSN. Table 5 shows a summary of all the variations of each parameter to be modified in the TSCH simulations. The combination of all these configuration parameter variations gives a total of 24 simulated configuration variations, and 30 iterations were performed for each of them.

**Figure 4:**
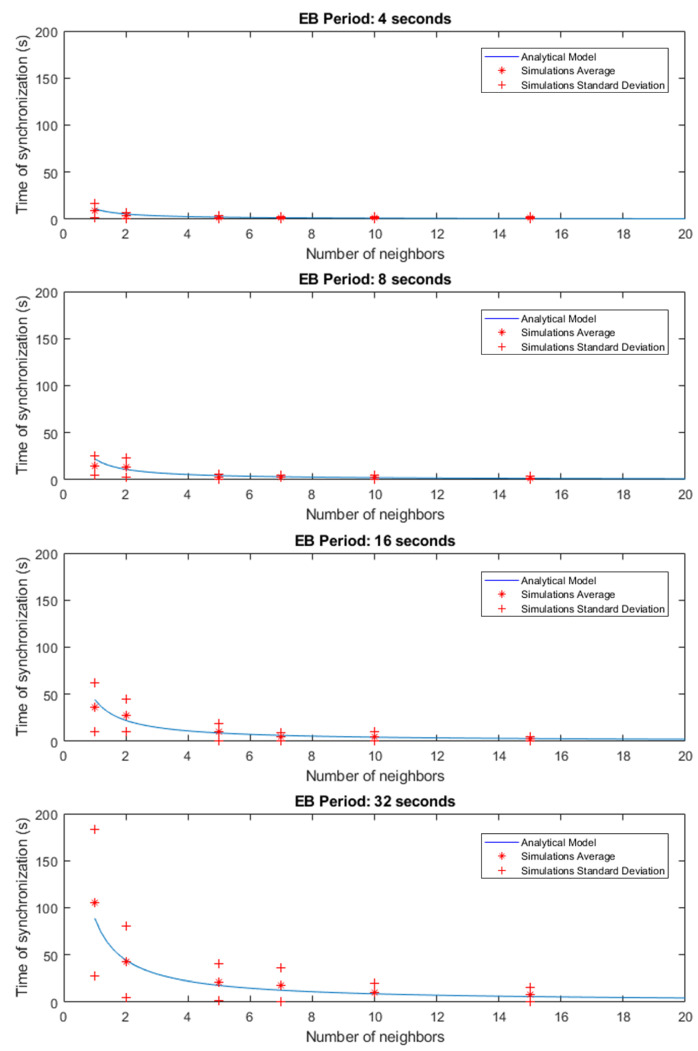
TSCH simulation results.

Figure 4 shows the results obtained for the TSCH simulations. The equation obtained from the TSCH analytical model is shown as a curve, superimposing the results of the average, using an asterisk, and standard deviation, with a plus sign, of the results of 30 simulations. As shown in the graphs, the simulations performed yielded results very close to the behavior of the proposed analytical model. From these results it is worth noting the behavior of the standard deviation of the results, whose dispersion is more accentuated in those simulations where the TSCH signaling traffic is quite dispersed. This occurs both in simulations where there are few nodes transmitting beacons and in those where the sampling period is longer. The most extreme case occurs when there is only one node and the transmission period is 32 s.

For the RPL simulations, the number of nodes was modified, as in the TSCH simulations, along with the transmission period of the DIO messages. In this case, instead of using the Trickle Timer for RPL, a fixed transmission period was used for multi-cast DIO messages once the network has stabilized, so that all nodes have a standardized DIO period value in each simulation variant. This simplification has been made due to the fact that the Trickle Timer is not synchronized between the nodes. In this way, a normalized value of the Trickle Timer is maintained in each simulation for all nodes, which allows analysis of each of the phases of the Trickle Timer period. In the case of the TSCH EB message transmission period, a fixed transmission period of 4 s was used to allow for rapid synchronization of the nodes. Table 5 also shows a summary of all the variations of each parameter to be modified in the RPL simulations. The combination of all these configuration parameter variations gives a total of 24 simulated configuration variations, and 30 iterations were performed for each of them.

**Figure 5:**
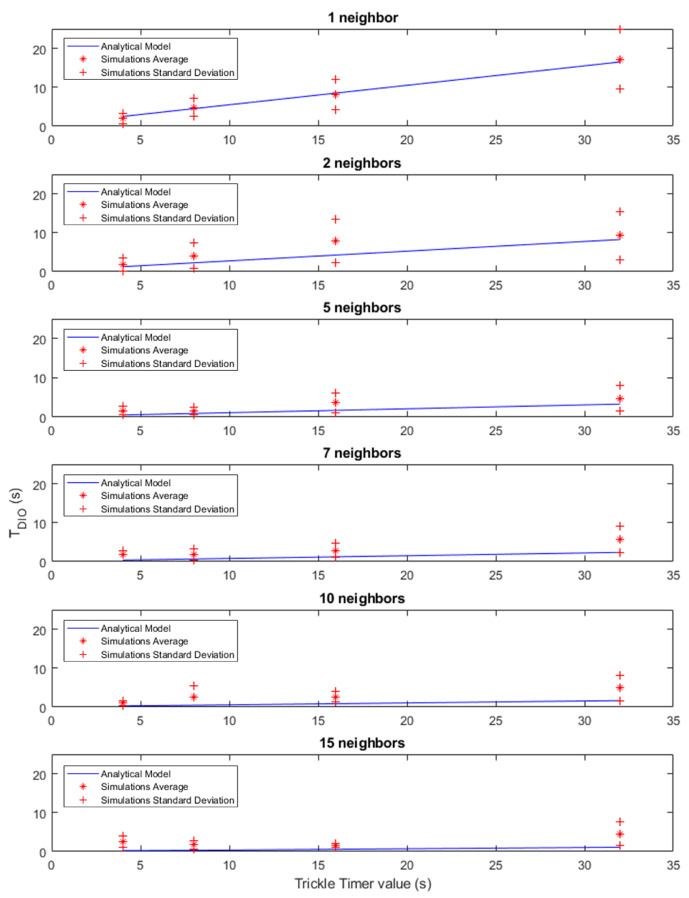
RPL simulation results.

Figure 5 shows the results obtained in the simulations carried out for the RPL part. The model and the results obtained have been represented using six different graphs depending on the number of neighbours, showing the results in the same way as in the case of the TSCH synchronization time. These graphs show how the time it takes the new node to obtain the information about the RPL tree increases in a linear way as the period between DIO messages increases, showing a higher dispersion of the standard deviation at the highest values of the period. It should be noted that the periodic sending of RPL signaling messages is not the normal operation of the protocol, but in stable situations and without events that reset the value of the Trickle Timer, the period between DIO messages would reach much higher values. Moreover the behavior between the different nodes would not necessarily follow the same pattern, since the state of the Trickle Timer is individual for each node.

However, through this individualized analysis, the impact that a certain period between DIO messages has on obtaining the information from the RPL tree can be demonstrated. The behavior of this message sending compromises between the traffic congestion produced by the RPL control plane and the speed at which a node is fully deployed and configured, so it is interesting to be able to control this behavior when deploying devices.

Another aspect to emphasize is the impact that the time that it takes to connect the node to the RPL tree has compared to the time that it takes the node to synchronize. Observing the scale of the graphs in Figure 4 and comparing them with the graphs in Figure 5, it is clear that the impact of the RPL part in the display and configuration of a node is lower than that of the TSCH synchronization time.

## 7. Real Testbed

As the last phase of this study, a battery of tests with real nodes was carried out, in order to check if the analytical model allows the value of the synchronization and connection time of a new node to the network to be characterized in an approximate way.

These tests have the same objective as the simulations, so the parameters to be modified in each of the protocols will be the same. However, given the duration of the real versus simulated tests, only a subset of these variations has been used. Table 6 shows the configuration parameters used during the real tests. As shown in the table, four different configurations were made with two period values of beacons and DIO messages and with two values for the number of nodes displayed.

The equipment used to carry out the deployment of the protocol stack based on Contiki was a RE-Mote from the manufacturer Zolertia. This equipment is based on the SoC CC2538, which is compatible with the IEEE 802.15.4 standard and with the TSCH media access mechanism, unlike its previous versions which were not compatible.

**Figure 6:**
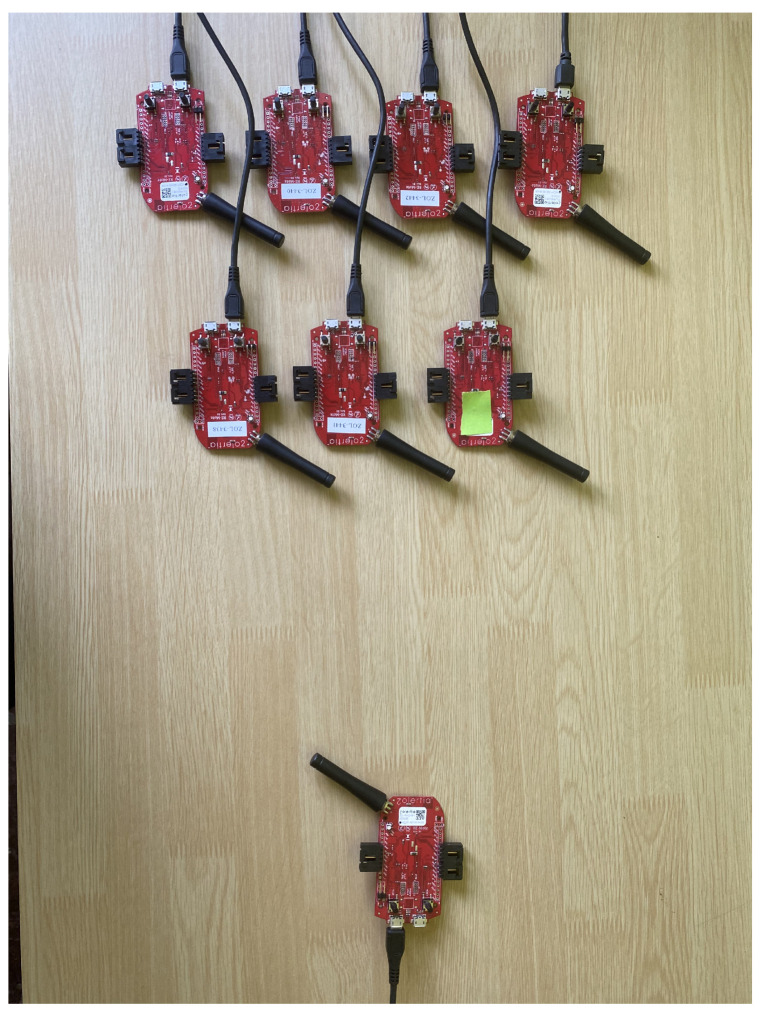
Testbed deployment using Zolertia RE-Mote platform.

In the procedure of the real tests the same pattern as in the simulations was followed. A node was configured that will do the work of the root of the sensor network, and will start both the beacon sending process and the DIO message transmission. The rest of the nodes will be synchronized from the information generated by this coordinating root node, depending on whether it is a two or seven-node deployment. Once the first node phase is displayed, the node to be characterized is turned on, detecting through a screen log the time instants in which the node starts, the instant in which it manages to synchronize when receiving an EB message and the instant in which it obtains the information from the RPL tree from the reception of a DIO message. This process was repeated a total of 15 times for each of the variations presented in Table 6. Figure 6 shows an image of the deployment made for the real tests.

**Figure 7:**
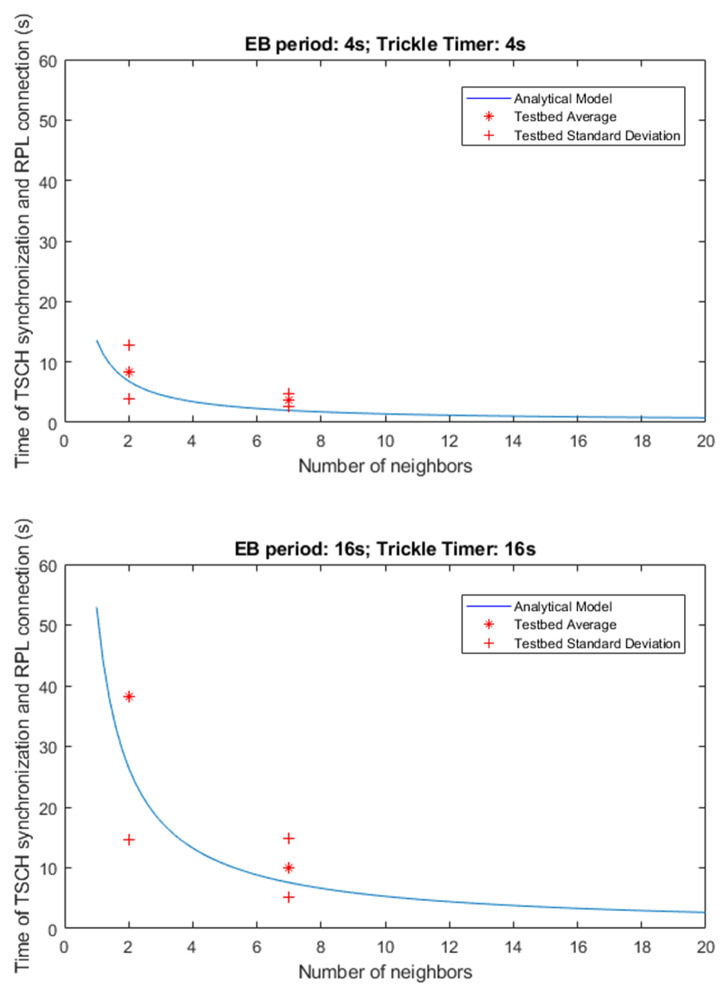
Testbed results—time of synchronization and RPL connection.

Figure 7 shows the results obtained during actual testing with RE-Mote devices. In this case, the graphs represent the sum of the total deployment and configuration time, which includes the time it takes to synchronize the new node and to obtain the configuration of the RPL tree. As mentioned above, the contribution of the TSCH part has a greater weight than that of RPL, so the shape of the curve is more similar to that of the TSCH analytical model with a Y-axis offset.

The results show how the values obtained for the total time of deployment of a new node fit the curve represented by the analytical model TSCH+RPL, so it gives a good estimate of the time required for a new node to synchronize and connect to a sensor network based on TSCH and whose routing mechanism is RPL. As happened in the simulations, the standard deviation dispersion is higher in those cases where the signaling traffic is considerably lower, and the case with more dispersion is that which uses a period of 16 s for the signaling traffic, both in TSCH and in RPL, and a number of nodes equal to 2.

## 8. Conclusions

This article has focused on the characterization of synchronization times of wireless sensor networks based on the TSCH medium access mechanism and the RPL routing protocol. The phases of TSCH synchronization, together with the process of joining the RPL topology, allow the full time it takes for a node to be fully operational to start the exchange of messages with the WSN network to be determined, including the synchronization and the creation of the routing tables.

For this purpose, an analytical model has been proposed to estimate the operation times of these two protocols, managing to estimate, in a simple way, an approximate value of the time it takes for a node to start its normal operation after deployment. Most articles focus on analyzing only the behaviour of TSCH synchronization, while in this article an analysis of the complete protocol stack oriented to industrial scenarios have been carried out. In this way, by using the model it is possible to estimate these operation start-up delay for certain types of applications. For applications that require a fast connection and deployment of the whole network, a low transmission period for TSCH beacons and RPL Trickle Timer must be configured. However, this causes increased energy consumption in the long term, so using dynamic beacon mechanisms would improve this compromise between a fast deployment and low energy consumption during network operating time.

The proposed analytical model has been validated through different simulations, and results were obtained that fit the behavior described by the model. In addition to the simulations, the model has also been validated through tests with real equipment, based on the CC2538 SoC wireless microcontroller, again with results that also fit the behavior described by the model.

From the results obtained, it has been proven that in a WSN network based on TSCH and RPL, the synchronization phase has a greater contribution to the total time it takes to deploy a node. However, these protocols are closely related because the Trickle Timer algorithm, which characterizes the sending of signaling traffic in RPL, can reach very high transmission periods during the synchronization phase. Thus, by speeding up the synchronization process, the RPL timer value is not at its maximum value, which also speeds up the formation of the RPL topology. Through the model proposed in this article, it is possible to determine which configurations are optimal in each phase of network operation and for different types of applications. The model defines configurations that allow the times of formation of the network during the phases of deployment to be favored, against configurations that are centered solely on maintaining the life-span of the network as long as possible.

## Figures and Tables

**Table 1:** TSCH analytical model variables.

Symbol	Description
$T_{EB}$	Represents the beacon transmission period.
$SF_{SIZE}$	Represents the size of the slotframe dedicated to the TSCH traffic plane, counted as the number of periodically recurring timeslots.
*C*	It represents the total number of channel used.
*N*	Represents the number of neighbors sending beacons within the coverage area of the node that wants to join the network.
$T_{SCAN}$	Scanning period. In TSCH, the channel is changed randomly every $T_{SCAN}$ seconds.
$\bar{SF}$	Represents the average number of slotframes needed to receive an EB correctly.
PDR	The Packet Delivery Rate parameter represents the message reception success rate.
$T_{SYNC}$	Represents the time needed for a new node to be synchronized, taking into account the number of neighbors and the beacon transmission period.

**Table 2:** RPL analytical model variables.

Symbol	Description
*P*	Probability of a node transmitting a DIO message.
$T_{trickle}$	Represents the period of transmission of DIO control messages.
$SF_{RPL}$	Represent the size of the slotframe dedicated to the RPL traffic plane, counted as the number of timeslots that are repeated periodically.
*N*	Represents the number of neighbors sending RPL control messages within the coverage area of the node that wants to join the network.
$t_{pdr}$	Represents the time required to send a DIO message based on the probability of the PDR parameter.
$T_{DIO}$	Represents the time needed to receive a DIO message after the node is synchronized to the TSCH network.
PDR	The Packet Delivery Rate parameter represents the success rate of message reception.

**Table 3:** Simulation configuration.

Parameters	Values
Number of neighbors for the new node	[1; 2; 5; 7; 10; 15]
Seeds per simulation	30
Time until stationary network	15–20 min

**Table 4:** Protocol stack configuration.

Parameters	Values
TSCH Scan Duration	256 s
TSCH Timeslot Duration	10 ms
Orchestra Slotframe size for Ebs	101 slots
Orchestra Slotframe size for RPL	101 slots
TSCH Number of Channels	4 channels
TSCH Number of Scan Channels	4 channels
RPL Trickle Timer Interval Min	4 s
RPL Trickle Timer Interval Doublings	From 1 to 4
RPL Trickle Timer Redundancy Constant	10
RPL DIS Interval	60 s

**Table 5:** Simulation parameters for TSCH and RPL simulations.

EB Period in TSCH Simulations	Number of Nodes	DIO Period in RPL Simulations
4 s	1	4 s
8 s	2	8 s
16 s	5	16 s
32 s	7	32 s
-	10	-
-	15	-

**Table 6:** Real testbed configuration parameters.

EB Period	DIO Period	Number of Nodes
4 s	4 s	2
16 s	16 s	2
4 s	4 s	7
16 s	16 s	7

## Data Availability

Not applicable.
